# Returning to Paid Employment after Stroke: The Psychosocial Outcomes In StrokE (POISE) Cohort Study

**DOI:** 10.1371/journal.pone.0041795

**Published:** 2012-07-25

**Authors:** Maree L. Hackett, Nick Glozier, Stephen Jan, Richard Lindley

**Affiliations:** 1 Neurological and Mental Health Division, The George Institute for Global Health, The University of Sydney, Sydney, New South Wales, Australia; 2 Psychological Medicine, Brain and Mind Research Institute, Sydney Medical School, The University of Sydney, Sydney, New South Wales, Australia; 3 The George Institute for Global Health, The University of Sydney, Sydney, New South Wales, Australia; 4 Neurological and Mental Health Division, The George Institute for Global Health, The University of Sydney, Sydney, New South Wales, Australia; University of Münster, Germany

## Abstract

**Objectives:**

To determine which early modifiable factors are associated with younger stroke survivors' ability to return to paid work in a cohort study with 12-months of follow-up conducted in 20 stroke units in the Stroke Services NSW clinical network.

**Participants:**

Were aged >17 and <65 years, recent (within 28 days) stroke, able to speak English sufficiently to respond to study questions, and able to provide written informed consent. Participants with language or cognitive impairment were eligible to participate if their proxy provided consent and completed assessments on the participants' behalf. The main outcome measure was return to paid work during the 12 months following stroke.

**Results:**

Of 441 consented participants (average age 52 years, 68% male, 83% with ischemic stroke), 218 were in paid full-time and 53 in paid part-time work immediately before their stroke, of whom 202 (75%) returned to paid part- or full-time work within 12 months. Being male, female without a prior activity restricting illness, younger, independent in activities of daily living (ADL) at 28 days after stroke, and having private health insurance was associated with return to paid work, following adjustment for other illnesses and a history of depression before stroke (C statistic 0·81). Work stress and post stroke depression showed no such independent association.

**Conclusions:**

Given that independence in ADL is the strongest predictor of return to paid work within 12 months of stroke, these data reinforce the importance of reducing stroke-related disability and increasing independence for younger stroke survivors.

**Trial Registration:**

Australian New Zealand Clinical Trials Registry ANZCTRN 12608000459325

## Introduction

Reducing the increasing burden of stroke requires sustained, collaborative, evidence-based action [1]. However, the needs of those in paid employment at the time of their stroke are often overlooked [2]. This is surprising given that most OECD countries have seen a dramatic rise in the proportion of their working age population claiming early ill-health-retirement and associated income-replacement benefits [3], which total 3–5% of national GDP [4]. In 2011 the federal government of Australia announced major changes in access to the disability support pension eligibility to encourage more disabled people to remain in the workforce [5].

Early dislocation from the workforce commonly leads to significant personal and economic losses [6], [7]. Returning to paid work is a key recovery milestone for many stroke survivors [8]. Those who return have better long-term outcomes [2], [9]. Conversely, those who do not return are more likely to experience impairment in their family life, social participation, and finances [2]. Differing economic environments probably explain much of the wide variation that has been observed previously in the rate at which stroke survivors return to work, although this research is characterised by a lack of consistency in study methods, e.g. a lack of standardisation in definitions of work, return to work, low power and post hoc analyses [2], [10].

Studies in other clinical conditions have demonstrated the influence of depression on the likelihood of return to work [11] over and above clinical indicators. Factors such as patients' perceptions of their work being stressful, job satisfaction and private health insurance status (Australia has a universal free public hospital system and private health insurance is usually required to pay for extras such as ancillary care, elective procedures or admissions to a private facility. Supplementary private health insurance is held by 53% of Australians [12]) have been less systematically studied. Whilst return to work is more likely in those who have milder strokes [10], [13], [14], [15] we were particularly interested in determining whether modifiable psychosocial factors like depression, present in one third of stroke survivors [16], or economic factors, were associated with return to paid work. POISE (the Psychosocial Outcomes In StrokE study) was a prospective study of younger (<65 years of age) stroke survivors, designed specifically to determine if modifiable early (within 28 days of stroke) psychosocial factors (e.g. depression) are associated with return to work one year after stroke.

## Methods

The POISE methods have been described elsewhere [17]. POISE was a prospective observational cohort study. Consecutive participants were recruited from 20 general public hospitals in the Stroke Services New South Wales (SSNSW) clinical network in Australia between Oct 2008 and June 2010. Participants were included if aged over 17 years and less than 65 years, had a recent (within 28 days) stroke, were able to speak English sufficiently to respond to study questions, and they or their proxy were able to provide written informed consent which included consent to contact the participant's general practitioner (GP) if necessary. Participants with receptive aphasia, a severe language disorder or cognitive impairment, as determined by their clinician, were eligible to participate if their proxy provided consent and completed assessments on the participants' behalf. Consent was obtained by hospital-based clinical staff prior to hospital discharge.

Hospital-based staff completed screening logs for all potential participants and collected baseline demographic (name, date of birth, contact information, cognitive competence, general practitioner information) and stroke (date, subtype, Glasgow Coma Scale score, received recombinant tissue plasminogen activator) information for consented participants, entered into secure internet-based case report forms. All participants were interviewed over the telephone (or face-to-face when necessary) at baseline (28-days), six and 12 months after stroke by trained interviewers based at The George Institute for Global Health in Sydney.

During the telephone interviews data were also collected on depression (Hospital and Anxiety Depression Scale depression subscale score ≥8, HADS-D [18]), anxiety (HADS anxiety subscale score ≥8, HADS-A [18]), cognitive function (telephone interview for cognitive status, TICSm [19]), instrumental activities of daily living (Frenchay activities index, FAI [20]), ‘at risk’ alcohol consumption (alcohol use disorder identification test, AUDIT-C [21]), and fatigue (vitality domain of the short form 36 questionnaire, SF-36 [22]).

Information on paid work was collected using modified versions of questions 34–51 of the *Australian Bureau of Statistics 2006 Census*. Paid work was defined as any type of work in the month before stroke, including casual or temporary, for one hour or more for which some form of payment was received. Unpaid work was grouped as follows: unpaid domestic work for the household; unpaid care, help or assistance for a family member or others because of a disability; long term illness or problems due to ageing; looking after a own child(ren) without pay; looking after someone else's child(ren) without pay; and voluntary work; graded as <5 hours, 5–14 hours; 15–29 hours and 30 hours or more per week. Participants indicated the age they expected to retire before their stroke, whether, and in what capacity, they had returned to work, and the date of return. Participants not returning to work were asked if they still wanted to return. Psychosocial barriers to return to work were determined using the short form of the job content questionnaire (JCQ [23]). High job stress was defined as those jobs with high demands (e.g. long hours) and low control (e.g. limited or no choice concerning what work was completed). Economic factors included health and income protection insurance.

Sample size was determined assuming a prevalence of depression of 25% and an overall return to paid work of 50%, as in previous Australasian studies [14]. To detect a relative risk of returning to work of 0·5 amongst the depressed we required 150 participants. We aimed to recruit 220 participants in paid, and 220 in unpaid work before stroke to allow for loss to follow-up, potential clustering effects (participants from the same hospital may be more similar to each other than participants in other hospitals), missing data and to provide sufficient numbers for separate multivariate models of return to paid and unpaid work.

Complete participant analyses were conducted using SAS version 9·2 [24] following the published analysis plan [17]. Potential predictors of the categorical primary end-point (returned to paid work) were identified using univariate analyses (p-value<0·20). Correlation and first order interaction between variables was assessed. One multivariable logistic regression model was built using manual variable selection of all variables significant at p<0·20, a second using backward elimination of all such variables, both with adjustment for known predictors of good stroke outcome. Models were compared using log-likelihood tests and area under the curve (AUC). The predictive ability of the final model was assessed by AUC using bootstrap methods to reduce bias, and sensitivity analyses were conducted to determine the impact of missing data.

Ethical approval was received from the Human Research Ethics Committee of the Sydney South West Area Health Service in May 2008, protocol X08-0084.

## Results

**Figure 1:**
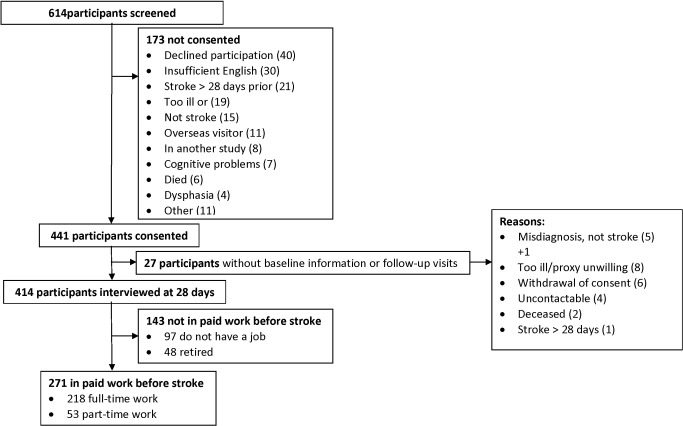
Flow of participants in POISE.

618 patients were screened for participation with only 40 directly declining to participate (see Figure 1). 441 participants were recruited and by the 28-day baseline interview 25 (6%) participants had withdrawn from the study. The remaining participants who were in full-time (218, 52%) and part-time paid employment (53, 13%) immediately before their stroke comprised the study group (271, 65%). Compared with those not in paid employment before stroke, the study group were younger (51±10 vs. 55±10 years of age, p = 0·002), more likely to be male (72% vs. 59%, p = 0·004), married/de facto (68% vs. 57%, p<0·05), had financially dependent children (39% vs. 23%, p<0·05), attained higher education qualifications (diploma/degree 41% vs. 27%, p = 0·007), were the main income earner (74% vs. 31%, p<0·001), had private health insurance (53% vs. 32%, p<0·001), were non-smokers (62% vs. 49%, p = 0·009), had no illness that restricted activity before stroke (83% vs. 67%, p<0·001), had no other illnesses (58% vs. 34%, p<0·001), and no prior depression (70% vs. 52%, p<0·001). The groups did not differ significantly on the proportion living alone or drinking heavily before stroke or by stroke subtype, Glasgow coma scale score, cognitive impairment, or the number receiving thrombolysis after stroke.

Table 1 shows the socio-demographic, clinical, and work profile of the study group. 202/271 (75%) participants returned to part- or full-time paid work during the first year. Most took 2–3 months to return and returned to the same part- or full-time job they had before stroke (see Table 2). Few employers made changes to the workplace, whereas 37% changed the work schedule or provided training or assistance for those when they first returned to work increasing to 48% over the first year after stroke. Only 7/271 (3%) participants had received job training, job-related counselling or job placement to aide a return to work at 28 days with that number rising to 20 (7%) over the first year. Variables significantly associated with return to work in the univariate analyses were higher education level (OR 2·16, 95% CI 1·12 to 4·14); being self-employed (OR 2·28, 95% CI 1·07 to 4·87); having a non-manual job (OR 2·06, 05% CI 1·18 to 3·61); being either independent in activities of daily living (OR 10·24, 95% CI 4·94 to 21·23) or free of depression (OR 2·41, 95% CI 1·07 to 5·42) at 28 days after stroke.

**Table 1:** POISE study group characteristics by return to paid work in the first year after stroke (univariates).

	Returned to paid work†				
	(n = 271)				
	Yes (n = 202)	No (n = 69)	Odds ratio	95% confidence interval	p-value
***Before stroke***					
Male	149/202 (74%)	47/69 (68%)	1·32	0·73 to 2·39	0.37
*Age, mean (±SD)	50·7 (10·1)	52·9 (9·3)	0·98	0·95 to 1·01	0.11
*Education					
School certificate or less (ref)	57/201 (28%)	29/69 (42%)	1		
HSC/trade certificate	55/201 (27%)	19/69 (28%)	1·47	0·74 to 2·93	0.27
*Diploma/degree	89/201 (44%)	21/69 (30%)	2·16	1·12 to 4·14	0.21
*Marital status					
Never married	30/202 (15%)	5/69 (7%)	2·67	0·87 to 8·13	0.08
Married/defacto	136/202 (67%)	48/69 (70%)	1·26	0·64 to 2·47	0.50
Other (ref)	36/202 (18%)	16/69 (23%)	1		
Lives in rural area	33/202 (16%)	11/69 (16%)	1·03	0·49 to 2·17	0.94
Lives with others	29/202 (14%)	10/69 (14%)	1·01	0·47 to 2·20	0.98
Number of financially dependent children, mean (±SD)	0·8 (1·1)	0·7 (1·1)	1·02	0·80 to 1·31	<0.01
Main income earner	153/201 (76%)	47/68 (69%)	1·42	0·78 to 2·62	0.25
Current non-smoker	128/202 (63%)	40/69 (58%)	1·25	0·72 to 2·19	0.43
Not heavy drinker	174/202 (86%)	59/69 (86%)	1·05	0·48 to 2·30	0.90
No illness that restricted activity before stroke	169/202 (84%)	54/68 (79%)	1·33	0·66 to 2·66	0.42
No other comorbid illnesses	115/202 (57%)	41/68 (60%)	0·87	0·50 to 1·52	0.63
No history of depression	141/202 (70%)	49/69 (71%)	0·94	0·52 to 1·72	0.85
Frenchay Activities Index, mean (±SD)	31·5 (6·2)	32·5 (7·1)	0·98	0·93 to 1·02	<0.01
***Work***					
Worked full-time	168/202 (83%)	50/69 (72%)	1·88	0·99 to 3·58	0.06
*Self employed/own business	53/115 (46%)	12/44 (27%)	2·28	1·07 to 4·87	0.03
*Non-manual job	114/198 (58%)	27/68 (40%)	2·06	1·18 to 3·61	0.01
Union member	55/173 (32%)	16/54 (30%)	1·11	0·57 to 2·16	0.76
*Job strain index					
Hi demands/low control (high job stress)	42/202 (21%)	21/69 (30%)	0·66	0·34 to 1·27	0.21
Low demands/high control	57/202 (28%)	14/69 (20%)	1·34	0·67 to 2·71	0.41
Others (ref)	103/202 (51%)	34/69 (49%)	1		
No health insurance	86/202 (43%)	40/68 (59%)	0·52	0·30 to 0·91	0.02
No income protection insurance	168/201 (84%)	58/68 (85%)	0·88	0·41 to 1·89	0.74
*Years in current job, mean (±SD)	12·1 (11·2)	15·6 (13·9)	0·98	0·96 to 1·00	<0.01
***Up to 28 days post stroke***					
Stroke subtype					
Intracerebral haemorrhage (ref)	24/202 (12%)	9/69 (13%)	1		
Subarachnoid haemorrhage	1/202 (1%)	0/69 (0%)	-	-	-
Ischaemic stroke	168/202 (3%)	57/69 (83%)	1·11	0·49 to 2·52	0.81
Unknown subtype	9/202 (4%)	3/69 (4%)	2·63	0.28 to 24.44	0.40
Received thrombolysis	11/200 (6%)	6/69 (9%)	0·61	0·22 to 1·72	0.35
Received job training/counselling/placement	5/202 (2%)	2/69 (3%)	0·85	0·16 to 4·49	0.85
*Independent in activities of daily living	181/196 (92%)	33/61 (54%)	10·24	4·94 to 21·23	<0.01
Cognitively competent at 28 days	167/195 (86%)	41/52 (79%)	1·60	0·74 to 3·48	0.24
*Not depressed at 28 days (HADS-D<8)	175/195 (90%)	40/51 (78%)	2·41	1·07 to 5·42	0.03

Numbers are n (%) unless otherwise specified;

* p<0·20 in univariate analyses; ref = reference group in univariate analyses; HADS-D hospital anxiety and depression scale depression subscale score; HAD-A hospital anxiety and depression scale anxiety subscale score.

† 76 (38%) of the participants who returned to paid work had done so by their baseline visit.

**Table 2:** Work situation for those who returned to paid work in the first year after stroke.

	Full-time paid work before stroke	Part-time paid work before stroke
	(n = 168)	(n = 34)
Returned to full-time work	119 (71%)	2 (6%)
Returned to part-time work	49 (29%)	32 (94%)
Number of days to return to work, mean (±SD)	65 (69)	86 (85)
Same employer		
Same job	143 (85%)	28 (82%)
Similar job	18 (11%)	1 (3%)
Different job	3 (2%)	0 (0%)
Changes made to workplace	9 (5%)	2 (6%)
Schedule changed, training or assistance given	62 (37%)	13 (38%)
Different employer		
Same job	0 (0%)	0 (0%)
Similar job	1 (1%)	0 (0%)
Different job	3 (2%)	5 (15%)

Numbers are n (%) unless otherwise specified; SD = standard deviation.

Among those who did not return to paid work, the proportion who wanted to return decreased over time from 87% (54/62) at 28 days to 66% (41/62) by 12 months. Before stroke these participants had expected to retire in 8·4 (SD 8·3) years on average (median 6 years, range −2.7 to 31.7 years [3 participants had worked past their expected retirement age]).

There was high correlation between HADS-D and HADS-A cases (Pearson correlation coefficient = 0·605) and a significant interaction between sex and presence of a prior activity restricting illness. Therefore only HADS-D was entered into the model and we generated composite variables for sex and ‘a prior activity restricting illness’. As models generated using the two methods were not statistically different the more parsimonious backwards elimination multivariate model is presented in Table 3.

**Table 3:** Final multivariable model showing variables associated with return to paid employment in the first year after stroke.

Covariate	Odds ratio	95% confidence interval
Female without illness that restricted activity before stroke	5·89	1·21 to 28·70
Male without illness that restricted activity before stroke	6·40	1·46 to 28·03
Male with illness that restricted activity before stroke	8·92	1·39 to 57·02
Female with illness that restricted activity (ref)	1	
Age, years	0·94	0·90 to 0·98
Education		
School certificate or less (ref)	1	
HSC/trade certificate	1·52	0·59 to 3·89
Diploma/degree	1·69	0·66 to 4·36
Main income earner	1·88	0·78 to 4·56
No other co-morbid illnesses	0·58	0·26 to 1·29
No history of depression	0·58	0·23 to 1·45
No health insurance	0·40	0·18 to 0·89
Not depressed at 28 days (HADS-D<8)	2·31	0·87 to 6·12
Independent in activities of daily living at 28 days	10·23	4·11 to 25·46

N = 240; C-statistic = 0·805; goodness of fit (Hosmer and Lemeshow goodness of fit test) p = 0·404.

Being self employed/working in own business excluded from final model due to large number of missing data (not significant when included).

**Figure 2:**
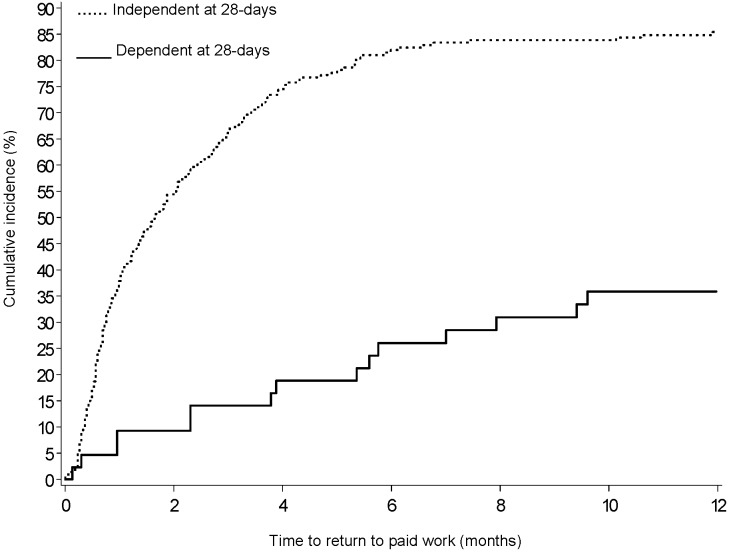
Cumulative incidence (Kaplan Meier plot) in time to return to work over one year by dependence of daily living status at 28 days.

**Figure 3:**
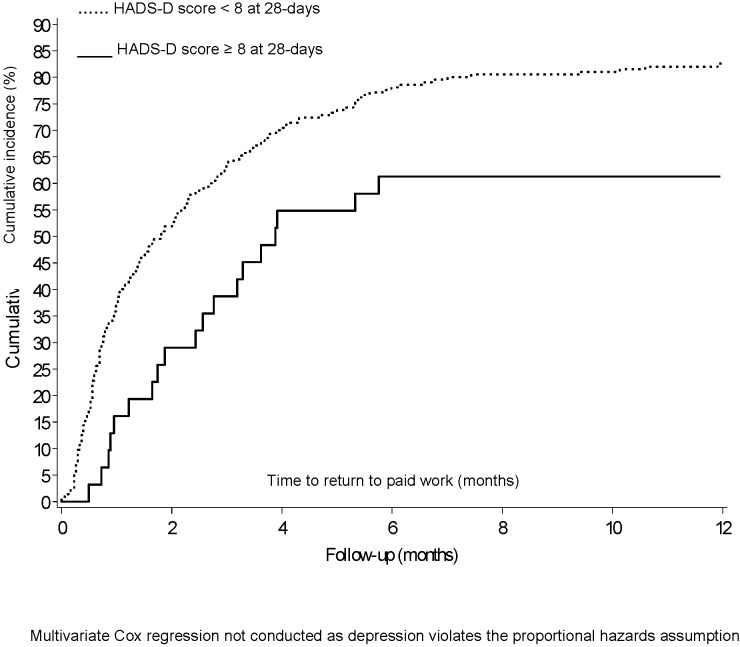
Cumulative incidence (Kaplan Meier plot) in time to return to work over one year by depression status at 28 days.

Those most likely to return to work in the 12 months following their stroke were independent in activities of daily living at 28 days after stroke (OR 10·23, 95% CI 4·11 to 25·46), had health insurance (not having health insurance OR 0·40, 95% CIs 0·18 to 0·89), were younger (OR 0·94, 95% CI 0·90 to 0·98), male, and female without a prior activity restricting illness. Although there was a reasonably strong effect size for not being depressed post stroke predicting greater return to work, this did not reach statistical significance (OR 2.31, 95% CI 0.87 to 6.12). The results of sensitivity analysis with multiple imputations for missing variables and the bootstrapping method to produce the confidence interval for the AUC remained consistent with the original analysis. The unadjusted time to return to work Kaplan Meier plots are presented in Figure 2 for those dependent and independent in ADL at 28 days and for those depressed and not depressed at 28 days in Figure 3.

## Discussion

We found 75% of those in paid employment before stroke returned to paid work in the year following stroke, most within 2–3 months. Those most likely to return were male, female without a prior activity restricting illness, younger, had private health insurance and were independent in activities of daily living 28 days after stroke. Only the last two factors might be considered modifiable and targets for intervention. Several key variables were found not to be associated with return to work including early depression, living in a metropolitan area, experiencing high job stress, having income protection insurance, being self employed or the main income earner in the household.

Given the ageing population, the need to retain people in the paid workforce and the increasing age at which retirement pensions are provided, the high proportion of this population who returned to work is reassuring. However, there is ongoing need for improvement for those who do not return to paid work, particularly the 2/3 who expressed a desire to return, and for the 29% who transitioned from full- to part-time paid work after stroke. The usual age for this transition is 60 years [25], not 50 years as seen in this cohort. As the current eligible age for the government means-tested retirement-benefit in Australia is 65 years [25] this group will spend on average 15 years claiming some form of income support; most of those making such claims stay on this support until retirement age [26]. In response, addressing the low rate of vocational training (3%) by promoting uptake and increasing availability should be a focus of future intervention.

Although health insurance status was positively associated with return to work, it is unlikely that increasing health insurance coverage would lead to improved rates of return to work. This association is more likely due to health insurance status being a strong marker of socioeconomic status in Australia where tax breaks offered for private health insurance coverage increase markedly according to income. Indeed, for instance, only 23% of those in the lowest income quintile in Australia have private health insurance [25]. Health insurance status could thus be positively associated with an improved prospect of return to work because those with higher incomes, and thus propensity to take up health insurance, will have a greater financial incentive to recommence work.

We did not confirm the hypothesis that early depression after stroke is associated with an inability to return to work [14]. In this younger cohort only 31 (11%) participants met the study criteria for depression at 28 days, much lower than is seen in other cohorts [14], [16] and lower than the 25% used in our sample size calculation. As not being depressed at one month still showed a reasonably strong but not statistically significant effect on the likelihood of returning to work within a year (adjusted OR 2·31, 95% CI 0·87 to 6·12) similar to that observed in other studies [14], [16], it is likely this study was underpowered to definitively confirm or refute this hypothesis. These data also highlight the significant impact of acquired disability in people of working age after stroke, which may in turn cause depression, which is not seen after other acute cardiovascular diseases where the direct impact of depression and psychosocial factors is clear [11], [27].

The gender effect on return to work has been seen elsewhere in Australia where the impact of prior illness was more pronounced upon ill-health-retirement in women than men [28]. This raises the question of how to engage women with chronic illnesses in workforce participation when such policies and programmes may be biased towards male roles and expectations. For instance women are more likely to be in precarious short term employment [29] with few prospects of their job being held open after significant illness, and also report that their spouse's income is an important determinant of whether they carry on working [30].

These data come from a prospective study with a protocol published prior to data analysis [17]. Participants were from a wide geographical and ethnically diverse population attending a well-organised clinical network of stroke units in rural and metropolitan hospitals. The lower than expected depression rates may reflect increased recovery from a cohort recruited from such well organised care [31]. The cohort was limited to those surviving to 28 days after stroke making these findings particularly relevant for younger survivors without a devastating stroke. Despite this the age distribution of our younger population is similar to that found in Australian stroke incidence studies which include all strokes over a defined period [32], [33] with POISE participants constituting approximately 15% more people aged 35–54 years and 15% fewer in the 55–64 year bracket, The limitations of these data include the assessment of dependency in activities of daily living and depression at 28 days after stroke (after controlling for pre-stroke dependency and depression) when 38% of participants had already returned to work.

This study provides a more positive outlook than previous studies [2], [10] with three quarters of participants returning to paid work. However, an increased focus on initiatives to reduce post stroke disability [34] could still have a significant impact upon improving return to paid work for those who still wish to do so.
